# First-trimester maternal folate and vitamin B12 concentrations and their associations with first-trimester placental growth: the Rotterdam Periconception Cohort

**DOI:** 10.1093/humrep/deaf095

**Published:** 2025-05-15

**Authors:** M M van Vliet, S Schoenmakers, S P Willemsen, K D Sinclair, R P M Steegers-Theunissen

**Affiliations:** Department of Obstetrics and Gynaecology, Erasmus MC, University Medical Center, Rotterdam, The Netherlands; Department of Developmental Biology, Erasmus MC, University Medical Center, Rotterdam, The Netherlands; Department of Obstetrics and Gynaecology, Erasmus MC, University Medical Center, Rotterdam, The Netherlands; Department of Biostatistics, Erasmus MC, University Medical Center, Rotterdam, The Netherlands; School of Biosciences, Sutton Bonnington Campus, University of Nottingham, Leicestershire, UK; Department of Obstetrics and Gynaecology, Erasmus MC, University Medical Center, Rotterdam, The Netherlands

**Keywords:** one-carbon metabolism, folate, vitamin B12, placenta, pregnancy

## Abstract

**STUDY QUESTION:**

Are maternal folate and vitamin B12 concentrations associated with first-trimester placental growth?

**SUMMARY ANSWER:**

Maternal folate concentrations and commencement of folic acid supplements prior to conception, as compared to following conception, are positively associated with first-trimester placental volume (PV), whereas no associations were found for maternal vitamin B12 concentrations.

**WHAT IS KNOWN ALREADY:**

Besides the protective effect of folic acid supplement use against neural tube defects and other adverse birth outcomes, the preconceptional commencement of folic acid supplements is positively associated with postpartum placental size, although conflicting outcomes have been reported. Studies in mice show an association with vitamin B12 deficiency and decreased placental weight postpartum.

**STUDY DESIGN, SIZE, DURATION:**

Between January 2010 and December 2020, 480 pregnancies (727 longitudinal ultrasound measurements) with known maternal folate and/or vitamin B12 blood concentrations in the first trimester and 875 pregnancies (1430 longitudinal ultrasound measurements) with known timing of folic acid supplement initiation were included in the Rotterdam Periconception Cohort, a prospective, hospital-based observational cohort.

**PARTICIPANTS/MATERIALS, SETTING, METHODS:**

Red blood cell (RBC) folate and serum vitamin B12 concentrations were determined in first-trimester maternal blood, and the timing of folic acid supplement use was collected using validated questionnaires. PV was measured from serial 3-dimensional ultrasounds performed at 7, 9, and 11 weeks of gestation. Linear mixed models were used to assess the associations between maternal folate and vitamin B12 concentrations with first-trimester PVs. Analyses were adjusted for gestational age at ultrasound, maternal age, BMI, geographical background, education level, parity, smoking, mode of conception, and the other B vitamins. For validation, the association between the timing of folic acid supplement initiation (pre- or postconception) and PV was assessed.

**MAIN RESULTS AND THE ROLE OF CHANCE:**

The median RBC folate concentration was 1395 nmol/l (IQR 1169–1588) and the median serum vitamin B12 concentration was 314 pmol/l (IQR 241–391). For RBC folate, the smallest PVs were found in women in the lowest quartile, with the largest difference as compared to women in the fourth quartile: ^3^√PV (β = −0.141, 95% CI = −0.249 to −0.033, *P* = 0.010), corresponding to a 1.79 cm^3^ (−18.7%) and a 6.99 cm^3^ (−9.9%) smaller PV at 7 and 11 weeks of gestation, respectively. Additionally, PV was significantly smaller in women who initiated folic acid supplements following rather than prior to conception: ^3^√PV (β=−0.129, 95% CI = −0.207 to −0.051, *P* = 0.001) corresponding to a 1.69 cm^3^ (−16.9%) and a 6.62 cm^3^ (−8.9%) smaller PV at 7 and 11 weeks of gestation, respectively. We found no significant association between maternal serum vitamin B12 concentrations and PV.

**LIMITATIONS, REASONS FOR CAUTION:**

The observational design of this study does not exclude residual confounding, and our hospital-based study population, with mostly adequate RBC folate and serum vitamin B12 concentrations, could limit the generalizability of our results.

**WIDER IMPLICATIONS OF THE FINDINGS:**

Our results emphasize the importance of the preconceptional commencement of folic acid supplements to achieve adequate maternal RBC folate concentrations, which could support optimal placental growth during the first trimester and also protect against neural tube defects and other adverse birth outcomes.

**STUDY FUNDING/COMPETING INTEREST(S):**

This study was funded by the Department of Obstetrics and Gynecology and the Department of Developmental Biology of the Erasmus MC, University Medical Center, Rotterdam, the Netherlands. K.D.S. was in receipt of funding from the Biotechnology and Biological Sciences Research Council (BBSRC) (BB/K017810/1). The authors declare that they have no conflict of interests.

**TRIAL REGISTRATION NUMBER:**

NTR4356.

## Introduction

Folate and vitamin B12 are critical nutrients involved in one-carbon metabolism. Through the provision of methyl donors, one-carbon metabolism plays an essential role in cellular processes such as DNA synthesis and DNA methylation (Friso *et al.*, 2017). The periconceptional period is characterized by major epigenetic reprogramming, including the removal of most DNA methylation marks following conception, followed by a wave of remethylation in developing embryonic lineages (Steegers-Theunissen *et al.*, 2013; Greenberg and Bourc’his, 2019; Zeng and Chen, 2019). This influences both fetal and placental development, and necessitates an increased demand for methyl donors such as folate in pregnant women. Moreover, the use of periconceptional folic acid supplements reduces the prevalence of neural tube defects and is therefore recommended for women from preconception until at least the end of the first trimester (Wilson and O’Connor, 2022).

A well-functioning placenta is essential for fetal growth and development, and placental dysfunction is involved in the pathogenesis of various obstetric complications (Burton and Jauniaux, 2015). Although conflicting outcomes have been reported, several studies have found associations between low folate and vitamin B12 concentrations and adverse pregnancy outcomes such as preeclampsia and low birthweight (Behere *et al.*, 2021; Yu *et al.*, 2021; Kaldygulova *et al.*, 2023; Cui *et al.*, 2024; Finkelstein *et al.*, 2024). Folic acid has been shown to promote placental trophoblast invasion by affecting DNA methylation in placental tissues and cell lines (Williams *et al.*, 2011; Rahat *et al.*, 2022), and folate deficiency increases the apoptosis rate in human trophoblasts (Steegers-Theunissen *et al.*, 2000), while maternal vitamin B12 has been associated with the expression of angiogenic markers in the placenta (Mani *et al.*, 2020). Additionally, folate and vitamin B12 are involved in the remethylation of homocysteine to methionine, and can subsequently decrease homocysteine concentrations (Froese *et al.*, 2019; Kaldygulova *et al.*, 2023). Elevated homocysteine concentrations can impair endothelial function and have been associated with smaller first-trimester placental growth in our cohort as well as in pregnancies with placental-mediated complications (Gaiday *et al.*, 2018; Hoek *et al.*, 2021). Folate and vitamin B12 may thus also promote placental development by decreasing homocysteine concentrations.

A recent study showed a positive relationship between periconceptional folic acid use and measures of placental size postpartum but mixed results have been reported for placental weight (Bergen *et al.*, 2012; Fekete *et al.*, 2012). Likewise, no association was found between maternal vitamin B12 concentrations and postpartum placental weight in humans (Bergen *et al.*, 2012), but vitamin B12 deficiency has been suggested to impair placental development (Arcot *et al.*, 2024) and decreased placental weight has been found in mice fed a vitamin B12 deficient diet (Sapehia *et al.*, 2023). Previous studies have mainly investigated periconceptional folic acid use or first-trimester maternal folate and vitamin B12 concentrations in relation to postpartum placental growth. However, currently, placental growth can non-invasively be measured in the first trimester of pregnancy using 3-dimensional (3D) ultrasound. Studying periconceptional measures of folate/folic acid and vitamin B12 with first-trimester placental growth is less biased by a timing difference in measuring the outcome and exposure. We hypothesize that lower maternal blood concentrations of both folate and vitamin B12 are negatively associated with early serial placental volume (PV) measurements as a feature of placental growth. Therefore, the aim of this study was to investigate, in the first trimester of pregnancy, the associations between maternal red blood cell (RBC) folate (an indicator of the long-term 2–4 months folate status) and serum vitamin B12 concentrations, and placental growth. For the purpose of validation, we also aimed to investigate the associations between first-trimester maternal serum folate concentrations (an indicator of 1–3 days short-term folate status) and the timing of folic acid supplement initiation (pre- or postconception), and placental growth. The results may further indicate how to optimize placental development in the future.

## Materials and methods

All data were collected from the Rotterdam Periconceptional cohort (Predict study). Ethical approval of this study was obtained by the Medical Ethics Committee from the Erasmus MC, Rotterdam, The Netherlands (MEC-2004-0227). The Predict study was prospectively registered (NL4115 Dutch Trial Register).

### Study population

The Predict study is an ongoing prospective cohort study conducted at the Department of Obstetrics and Gynecology of the Erasmus MC, University Medical Center, The Netherlands, since November 2010 (Steegers-Theunissen *et al.*, 2016; Rousian *et al.*, 2021). Women aged ≥18 years with singleton pregnancies before 10 weeks of gestation were eligible for participation. Both parents signed a written informed consent form at enrollment.

### Study parameters

Participant characteristics were obtained through validated self-reported questionnaires addressing topics including medical and obstetrical history, education, and lifestyle behaviors, such as smoking, alcohol use, and the use of folic acid. Dietary intake was obtained using a validated food frequency questionnaire (FFQ) in the first trimester, from which dietary folate intake was determined using the Dutch food composition table by Wageningen University (Voortman *et al.*, 2020). Weight and height were measured at study entry by a research nurse. For naturally conceived pregnancies in women with a regular cycle between 25 and 31 days, gestational age was dated based on their first day of their last menstrual period (LMP). In case of an irregular cycle or unknown LMP, pregnancy dating was based on the crown-rump length (CRL) measured at the ninth-week ultrasound. If the gestational age calculated using the LMP differed by ≥6 days from that calculated based on the CRL, dating was based on the CRL. Pregnancies conceived after intrauterine insemination were dated based on the insemination date and were considered naturally conceived. The date of embryo transfer minus the number of days the embryo was cultured, was used to determine the conception date used to date pregnancies following IVF or ICSI.

### Folate and vitamin B12

A non-fasting blood sample was taken at study inclusion during the first trimester. For pregnancies included between November 2010 and December 2016, maternal RBC folate and serum folate and vitamin B12 concentrations were determined. The maternal RBC folate concentration was calculated using an in house developed protocol in which the hemolysate folate was corrected for hematocrit and the serum folate concentration. Both hemolysate and serum folate as well as total vitamin B12 concentrations were determined using an electrochemiluminescent immuno assay on a Cobas E801 analyser (Roche Diagnostics, Switzerland).

### PV measurement

**Figure 1.**
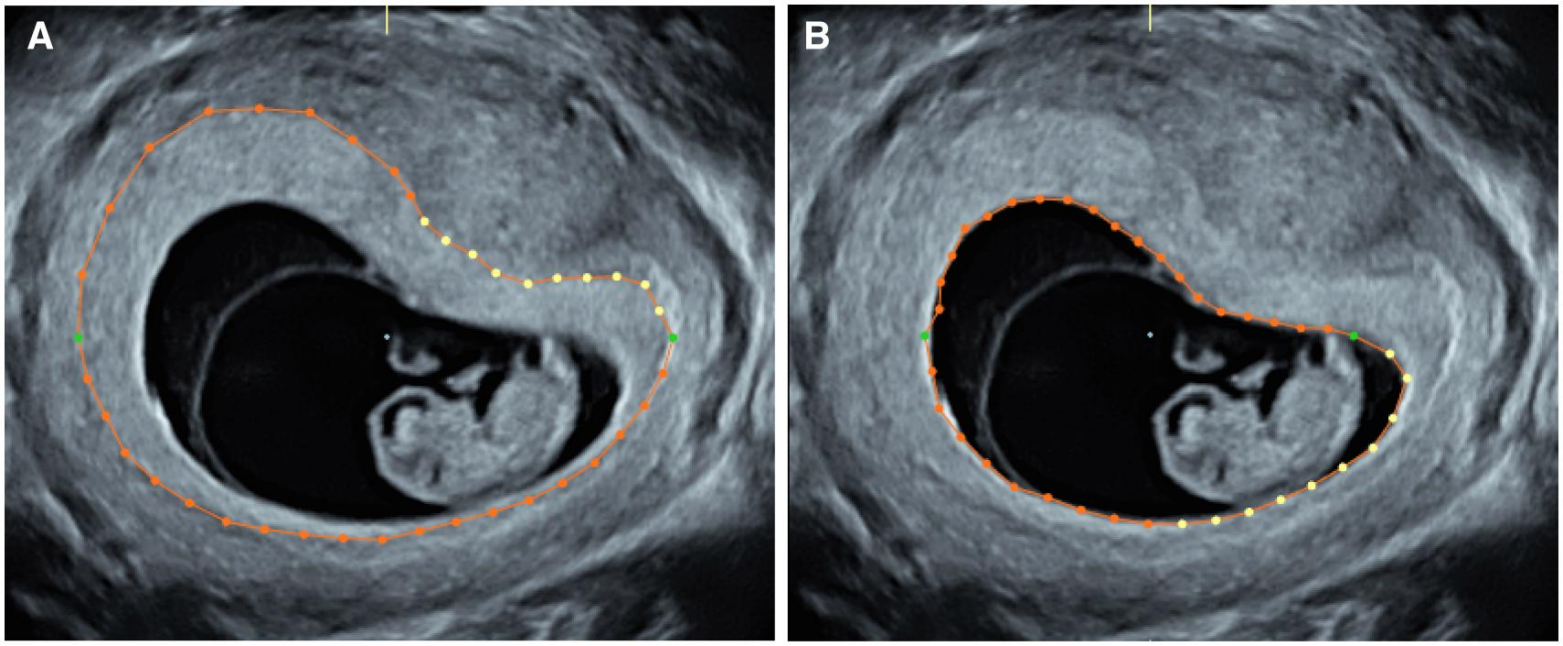
**Measuring placental volume (PV) using 3-dimensional ultrasound volumes.** The PV is calculated by the total pregnancy volume (**A**) minus the volume of the gestational sac (**B**).

The PV was measured at 7, 9, and 11 weeks of gestation using 3D ultrasonography to assess volumes of the whole pregnancy. These measurements were performed with standardized settings as described previously using a 6–12 MHz transvaginal probe compatible with the GE Voluson E8 Expert system (Reijnders *et al.*, 2021). PV was measured using Virtual Organ Computer-aided AnaLysis software as described previously (Reijnders *et al.*, 2018). In short, the placental outline (Fig. 1A) was repeatedly traced in rotational step of 15° to calculate the total pregnancy volume. Similarly, the contours of the gestational sac (Fig. 1B) were traced to establish the volume of the gestational sac, which was subsequently deducted from the total pregnancy volume to obtain the PV (Fig. 1).

### Statistical analysis

We excluded pregnancies in case of miscarriage, termination of pregnancy, intrauterine fetal death or study withdrawal. In cases where women participated in the Predict study across multiple pregnancies, only the first pregnancy was included in the analyses. Parental characteristics were described using medians and interquartile ranges for continuous variables, and percentages were used for categorical variables. Differences in baseline characteristics between women who initiated folic acid supplement use prior to conception and those who initiated folic acid supplement use following conception were assessed using the chi-square test for categorical variables, and the Mann–Whitney *U* test for continuous variables. Linear mixed models were used to assess the associations between maternal RBC folate and serum vitamin B12 concentrations and longitudinal measurements of PV. In addition to linear models, maternal folate and vitamin B12 concentrations were divided into quartiles and the lowest quartiles (Q1s) were used as a reference. Because of the skewed distribution of PV, a third root transformation was applied to the PV data.

For the linear mixed models investigating associations between maternal folate and vitamin B12 concentrations with PV, we first adjusted only for a cubic spline function of gestational age at the time of the ultrasound (model 1). In model 2, we additionally adjusted for fetal sex, parity, maternal age, maternal BMI, mode of conception, education level, geographical origin, and periconceptional smoking status. For validation, we investigated the association between serum folate and PV. Additionally, we investigated the relationship between the timing of folic acid supplement initiation (pre- or postconception) and RBC folate concentrations using the Mann–Whitney *U* test, and we investigated the association between the timing of folic acid supplement initiation and PV using linear mixed models with the same covariates as used in model 1 and model 2. For the association between the timing of folic acid supplement initiation and PV, we performed a sensitivity analysis additionally adjusting for dietary folate intake obtained through the FFQ. Only women with a total daily energy intake above the Goldberg cut-off (Black, 2000) were included, to exclude participants with unrealistic low intake of energy. Furthermore, we adjusted for RBC folate concentrations when analyzing the association with serum vitamin B12, and for serum vitamin B12 when studying the association with RBC or serum folate. *P*-values ≤0.05 were considered statistically significant and all analyses were performed using SPSS version 28.0.1.0 (142) (IBM SPSS Statistics, Armonk, NY, USA) and R (Version 4.3.2).

## Results

### Study population

**Figure 2.**
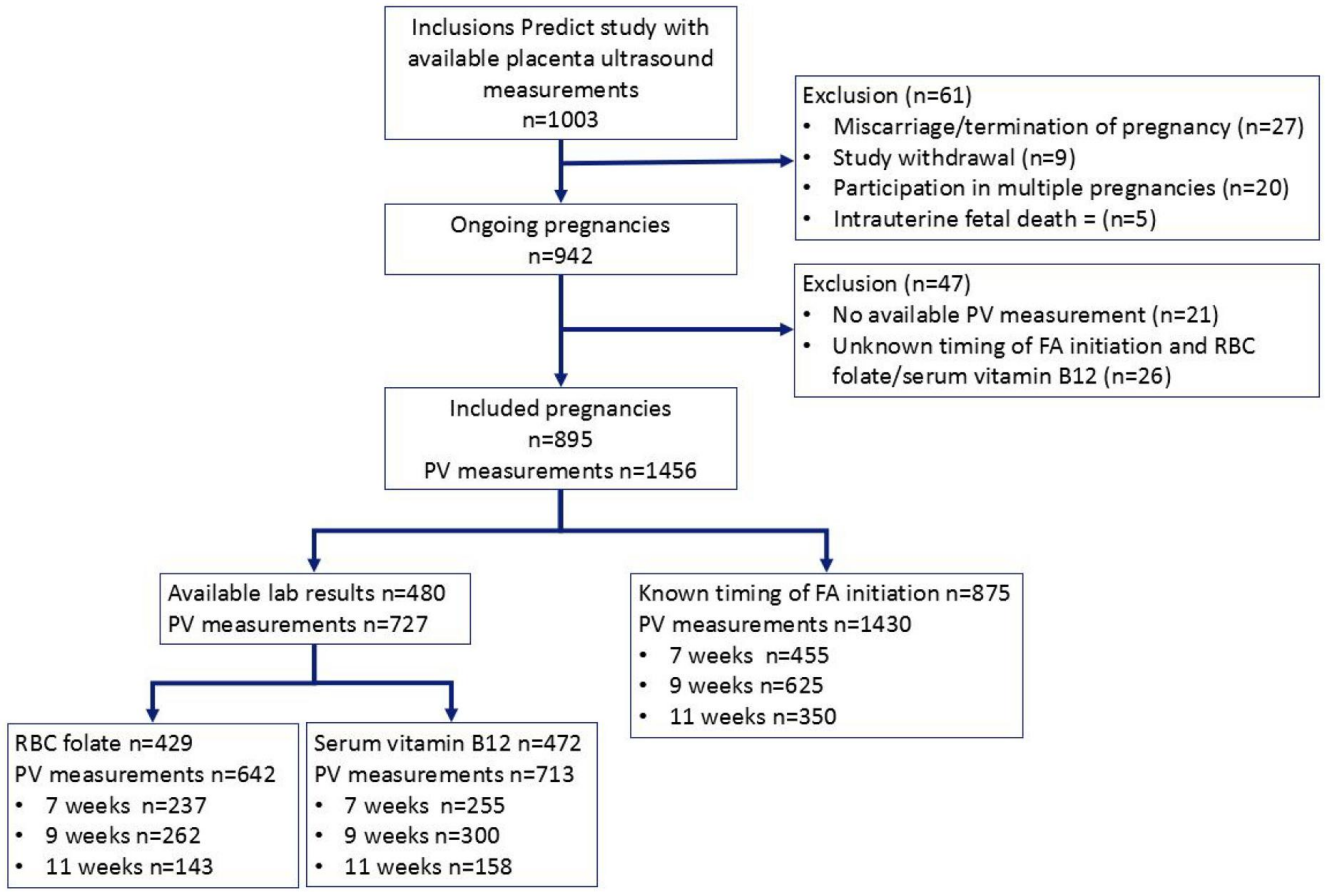
**Flowchart of the included study population.** RBC, red blood cell; PV, placental volume; FA, folic acid.

There were 1003 pregnancies with available ultrasound data included in the Predict study between November 2010 and December 2020. We excluded 108 pregnancies prior to analysis because of: miscarriage or termination of pregnancy (n = 27), study withdrawal (n = 9), participation in multiple pregnancies (n = 20), intrauterine fetal death (n = 5), no available PV measurement (n = 21), or no available RBC folate and serum vitamin B12 concentrations and unknown timing of folic acid supplement initiation (n = 26); in case women participated in the Predict study during multiple pregnancies, only the first pregnancy was included (exclusion of n = 20). Of the 895 remaining pregnancies, RBC folate concentrations were available for 429 pregnancies (642 PV measurements), serum vitamin B12 concentrations were available for 472 pregnancies (713 PV measurements), and the timing of folic acid supplement initiation was known for 875 pregnancies (1430 PV measurements) (Fig. 2).

### Periconceptional maternal characteristics

Baseline characteristics of included pregnancies with available RBC folate and/or serum vitamin B12 concentrations (n = 480) are depicted in Table 1. Mean maternal age of this study population was 32.0 years (IQR 28.8–35.1) and mean periconceptional maternal BMI was 24.5 kg/m^2^ (IQR 22.0–28.4). Our study population was predominantly of Dutch origin (79.6%) and highly educated (54.4%). Around half (56.3%) were nulliparous and 63.5% of pregnancies were conceived spontaneously. During the periconceptional period, 16.9% of women smoked and 32.9% used alcohol. For 98.3% of the study population, it was known that they used folic acid supplements during pregnancy, and 80.6% of women initiated folic acid supplements prior to conception.

**Table 1.** Baseline characteristics of included pregnancies with available red blood cell (RBC) folate and/or serum vitamin B12 concentrations (n = 480).

	Study sample (n = 480)
Age at conception	32.0 [28.8–35.1]
Geographical background	
Dutch	382 (79.6)
Western, other	26 (5.4)
Non-Western	58 (12.1)
Missing	14 (2.9)
Education level	
Low	36 (7.5)
Moderate	169 (35.2)
High	261 (54.4)
Missing	14 (2.9)
Nulliparous	270 (56.3)
Missing	8 (1.7)
Conception mode	
Spontaneous	305 (63.5)
IVF/ICSI	175 (36.5)
Body mass index (kg/m^2^)	24.5 [22.0–28.4]
Missing	3 (0.6)
Periconceptional smoking	81 (16.9)
Missing	11 (2.3)
Periconceptional alcohol use	158 (32.9)
Missing	11 (2.3)
Folic acid supplement use	472 (98.3)
Missing	8 (1.7)
Of which preconceptional initiation of folic acid supplements	387 (80.6)
Missing	20 (4.2)

Data are presented as median [interquartile range (IQR)] or n (%).

Baseline characteristics for women with known timing of folic acid supplement initiation (n = 875) are depicted in Table 2 and are largely comparable. Women who initiated folic acid supplements prior to conception were older, more often of Dutch origin, more highly educated, more often nulliparous, conceived more often after IVF/ICSI treatment, and smoked less often, as compared to women who initiated folic acid supplements following conception (all *P* < 0.001) (Table 2). Additionally, periconceptional maternal BMI was lower in women who initiated folic acid supplements prior to as compared to following conception (24.1 vs 25.5 kg/m^2^, *P* = 0.002). Dietary intake of folate assessed using the FFQ did not differ between groups.

**Table 2.** Baseline characteristics of included pregnancies based on timing of folic acid supplement initiation (n = 875).

	Total study population (n = 875)	Initiated FA preconceptional (n = 732)	Initiated FA postconceptional (n = 143)	*P*-value
Age at conception	32.2 [29.1–35.5]	32.5 [29.3–35.7]	30.1 [27.4–34.8]	<0.001*
Geographical background				<0.001*
Dutch	715 (81.7)	614 (83.9)	101 (70.6)	
Western, other	47 (5.4)	38 (5.2)	9 (6.3)	
Non-Western	112 (12.8)	80 (10.9)	32 (22.4)	
Missing	1	0	1	
Education level				
Low	65 (7.4)	40 (5.5)	25 (17.5)	<0.001*
Moderate	306 (35.0)	247 (33.7)	59 (41.3)	
High	502 (57.4)	443 (60.5)	59 (41.3)	
Missing	2	2	0	
Nulliparous	503 (57.5)	452 (61.7)	51 (35.7)	<0.001*
Missing	11	10	1	
Conception mode				
Spontaneous	497 (56.8)	364 (49.7)	133 (93.0)	<0.001*
IVF/ICSI	378 (43.2)	368 (50.3)	10 (7.0)	
Body mass index	24.2 [22.1–28.0]	24.1 [22.0–27.6]	25.5 [22.2–30.9]	0.002*
Missing	3	1	2	
Periconceptional smoking	130 (14.9)	88 (12.0)	42 (29.4)	<0.001*
Missing	1	1	0	
Periconceptional alcohol use	270 (30.9)	219 (29.9)	51 (35.7)	0.177
Missing	1	1	0	
Dietary folate intake (µg/day) (food frequency questionnaire)	262 [211–324]	263 [215–324]	255 [198–322]	0.306
Unknown or below Goldberg cut-off	**426**	**347**	**79**	

Data are presented as median [interquartile range (IQR)] or n (%). *Significance at *P* ≤ 0.05 assessed by chi-square test or Mann–Whitney *U* test as appropriate.

### Maternal folate and vitamin B12 concentrations and PVs

The median RBC folate concentration was 1395 nmol/l (IQR 1169–1588), and the median serum vitamin B12 concentration was 314 pmol/l (IQR 241–391) (Table 3). In our linear models, there were no significant associations between RBC folate or serum vitamin B12 concentrations and PV (Table 4). However, the quartile analyses show that for RBC folate, women in the lowest quartile had the smallest PVs (Table 5). Compared to women in the lowest quartile, PV was significantly larger in women with RBC folate concentrations in the second quartile or in the highest quartile in model 1 (Q2 ^3^√PV: β = 0.137 (95% CI 0.041–0.233), *P* = 0.005; Q4 ^3^√PV: β = 0.126 (95% CI 0.029–0.223), *P* = 0.011). Comparable results were found after adjustment for potential confounders in model 2 (Q2 ^3^√PV: β = 0.129 (95% CI 0.027–0.232), *P* = 0.014; Q4 ^3^√PV: β = 0.141 (95% CI 0.033–0.249), *P* = 0.010). For women with RBC folate concentrations in Q3 as compared to Q1, positive effect estimates were found for PV, although these were not statistically significant (model 1 ^3^√PV: β = 0.071 (95% CI −0.025 to 0.167), *P* = 0.147; model 2 ^3^√PV: β = 0.088 (95% CI −0.019 to 0.195), *P* = 0.107).

**Table 3.** First-trimester maternal red blood cell (RBC) folate and serum vitamin B12 concentrations in quartiles.

	RBC folate (nmol/l) (n = 429)	Serum vitamin B12 (pmol/l) (n = 472)
Q1	449–1169 (n = 108)	109–241 (n = 118)
Q2	1170–1395 (n = 107)	242–313 (n = 118)
Q3	1396–1588 (n = 107)	314–389 (n = 117)
Q4	1589–2919 (n = 107)	390–897 (n = 119)

**Table 4.** Effect estimates for associations between first-trimester maternal red blood cell (RBC) folate and serum vitamin B12 concentrations and first-trimester placental volumes ($\mathrm{PV}\;\sqrt[{3}]{{\mathrm{cm}}^{3}}$).

	RBC folate				Serum vitamin B12			
	Model 1		Model 2		Model 1		Model 2	
	Bèta (95% CI) (10^−3^)	*P*-value	Bèta (95% CI) (10^−3^)	*P*-value	Bèta (95% CI) (10^−3^)	*P*-value	Bèta (95% CI) (10^−3^)	*P*-value
**Continuous**	0.0901 (−0.0639 to 0.1866)	0.067	0.0948 (−0.0106 to 0.2002)	0.078	0.0861 (−0.1769 to 0.3491)	0.520	0.1102 (−0.1783 to 0.3987)	0.453

Model 1 is adjusted for gestational age at the ultrasound. Model 2 is additionally adjusted for fetal sex and maternal covariates age, geographic origin, education, periconceptional BMI, mode of conception, parity, and the other B vitamin.

**Table 5.** Effect estimates for associations between first-trimester maternal red blood cell (RBC) folate and serum vitamin B12 concentrations and first-trimester placental volumes ($\mathrm{PV}\;\sqrt[{3}]{{\mathrm{cm}}^{3}}$).

	**RBC folate**				Serum vitamin B12			
	Model 1		Model 2		Model 1		Model 2	
	Bèta (95% CI)	*P*-value	Bèta (95% CI)	*P*-value	Bèta (95% CI)	*P*-value	Bèta (95% CI)	*P*-value
**Q1**	Reference	Reference	Reference	Reference	Reference	Reference	Reference	Reference
**Q2**	0.137 (0.041–0.233)	0.005*	0.129 (0.027–0.232)	0.014*	−0.034 (−0.125 to 0.057)	0.461	−0.053 (−0.154 to 0.049)	0.308
**Q3**	0.071 (−0.025 to 0.167)	0.147	0.0879 (−0.019 to 0.195)	0.107	0.050 (−0.042 to 0.142)	0.282	0.011 (−0.093 to 0.116)	0.831
**Q4**	0.126 (0.029–0.223)	0.011*	0.1411 (0.033–0.249)	0.010*	0.033 (−0.058 to 0.124)	0.482	0.056 (−0.047 to 0.158)	0.286

Quartiles 1 are taken as reference. Model 1 is adjusted for gestational age at the ultrasound. Model 2 is additionally adjusted for fetal sex and maternal covariates age, geographic origin, education, periconceptional BMI, mode of conception, parity, and the other B vitamin.  *Significance at *P* ≤ 0.05.

For maternal serum folate concentrations, an indicator of short-term folate status, we again found the smallest PVs in women with the lowest serum folate concentrations. However, no significant associations were found for women with serum folate concentrations in Q2 or Q4 as compared to Q1 (Supplementary Table S1). However, compared to women with serum folate concentrations in the lowest quartile, PV was significantly higher in women with serum folate concentrations in the third quartile (model 1 ^3^√PV: β = 0.104 (95% CI 0.010–0.198), *P* = 0.030; model 2 ^3^√PV: β = 0.132 (95% CI 0.027–0.237), *P* = 0.014).

No significant associations were found between maternal serum vitamin B12 concentrations and PV (Table 5).

### Timing of folic acid supplement use initiation

**Figure 3.**
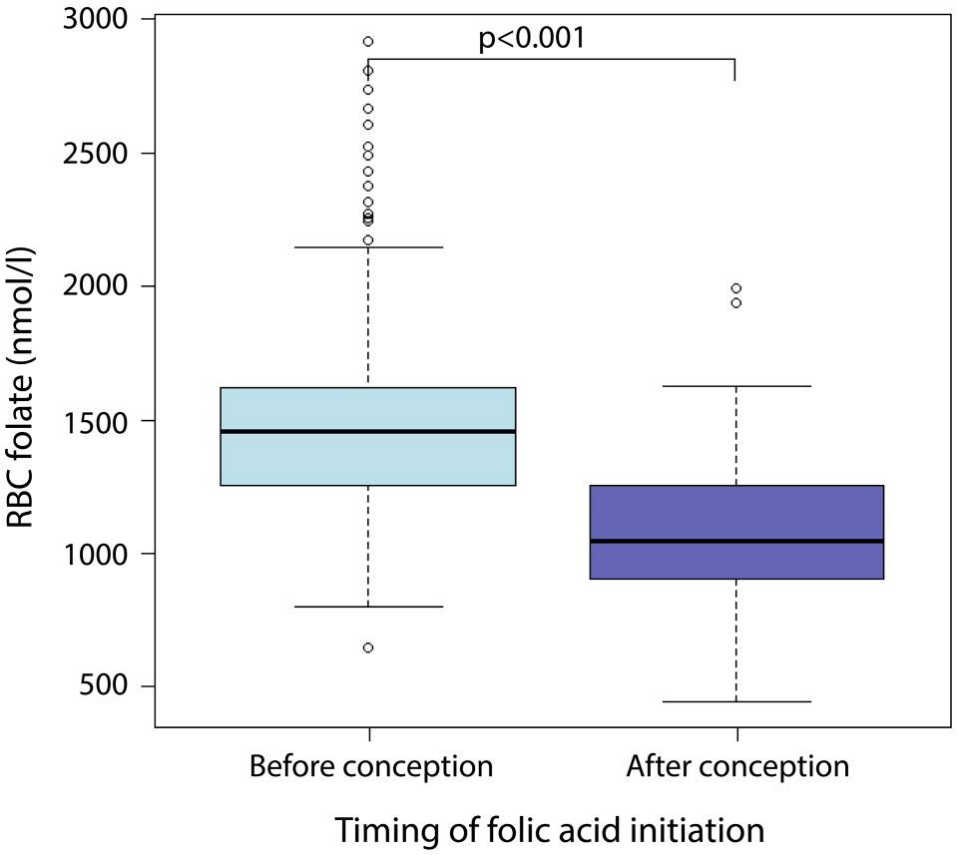
Red blood cell (RBC) folate (nmol/l) by timing of folic acid supplement initiation.

We assumed higher RBC folate concentrations in women who initiated the use of folic acid supplements prior to conception, compared to following conception. As expected, in our study population with known RBC folate concentrations, higher RBC folate concentrations were found in women who initiated folic acid supplement use prior to conception than in women who initiated folic acid supplement use following conception (1458 vs 1047 nmol/l, *P* < 0.001) (Fig. 3). Therefore, as a validation of the observed results for RBC folate concentrations, we investigated the association between the timing of folic acid supplement initiation (pre- or postconception) and PV in a larger cohort (n = 875).

As for lower RBC folate concentrations, PV was significantly smaller in women who initiated the use of folic acid supplements following conception, as compared to those who initiated folic acid supplements prior to conception (model 1 ^3^√PV: β=−0.117 (95% CI −0.185 to −0.049), *P* = 0.0007; model 2 ^3^√PV: β=−0.129 (95% CI −0.207 to −0.051), *P* = 0.0012). After additionally adjusting for dietary folate intake in a subset of pregnancies with reported energy intake (FFQ) above the Goldberg cut-off (n = 449, PV’s = 735), comparable results were found: ^3^√PV: β=−0.124 (95% CI −0.233 to −0.014), *P* = 0.027.

### Retransformation

**Figure 4.**
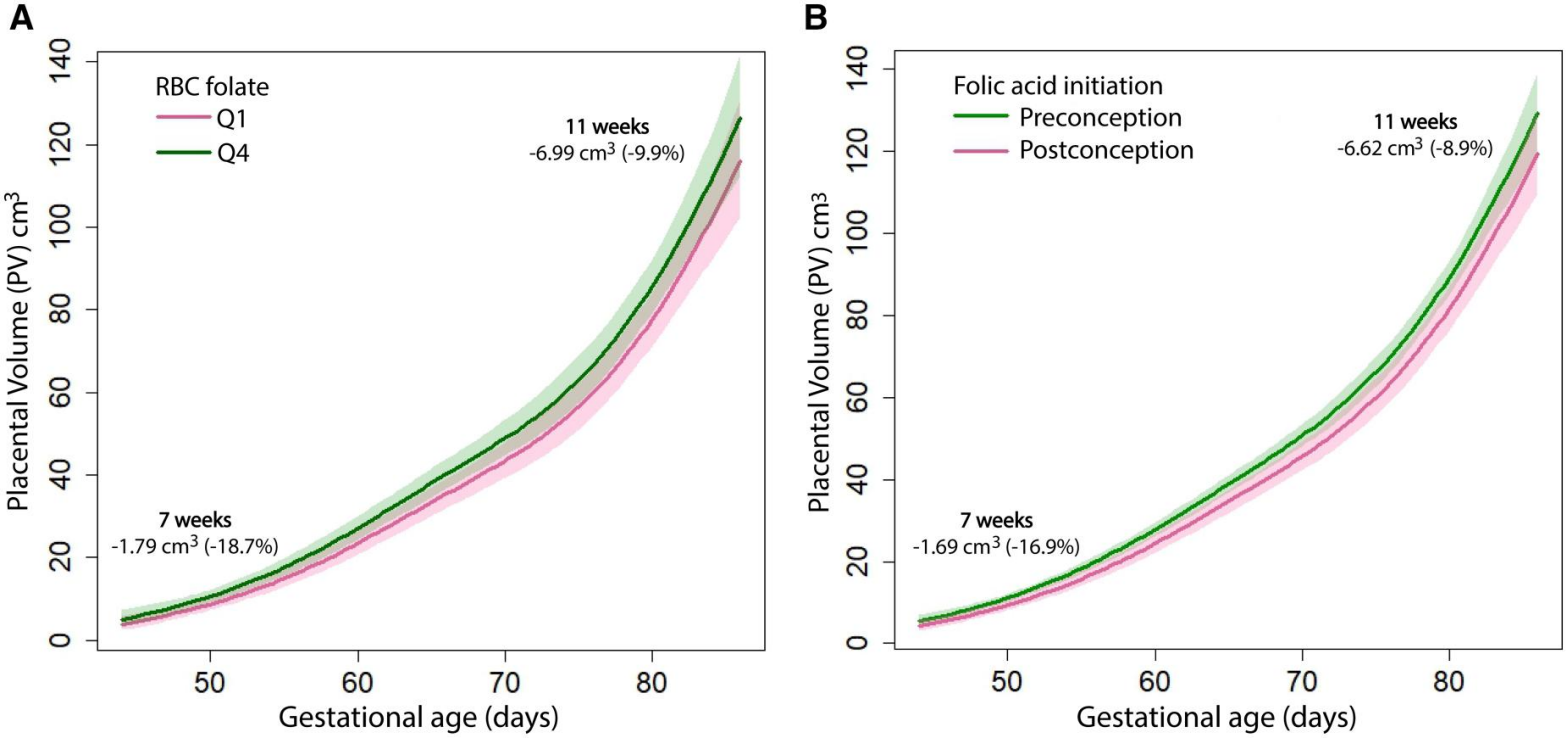
**Effect plots showing the placental volume (PV) trajectories during the first trimester of pregnancy.** (**A**) Red blood cell (RBC) folate Quartile 1 and Quartile 4. (**B**) Women who initiated the use of folic acid supplements prior to or following conception. Effect plots are based on model 2.

Retransformation of the effect estimates to the original scale shows that in women with RBC folate concentrations in Q1 as compared to Q4, PV was 1.78 cm^3^ (−18.7%) smaller at 7 weeks of gestation and 6.99 cm^3^ (−9.9%) smaller at 11 weeks of gestation in the fully adjusted model (model 2). For women who initiated folic acid supplements following conception, as compared to preconception, PV was 1.69 cm^3^ (−16.9%) and 6.62 cm^3^ (−8.9%) smaller at 7 and 11 weeks of gestation, respectively (model 2). Corresponding effect plots are shown in Fig. 4. Additionally, effect plots for the whole study population including individual data points by maternal RBC folate quartile and by timing of initiation of folic acid supplement use are depicted in Supplementary Fig. S1.

## Discussion

This study is the first to investigate the associations between maternal folate and vitamin B12 concentrations and first-trimester placental growth. We found smaller first-trimester PVs in pregnancies with the lowest maternal RBC folate concentrations. PV was 18.7% and 9.9% smaller at 7 and 11 weeks of gestation, respectively, in women in the lowest quartile compared to women in the highest quartile. These effect sizes were comparable to the smaller PVs found in women who initiated folic acid supplements following conception compared to women who initiated folic acid supplement use prior to conception. No significant associations were found between serum vitamin B12 concentrations and PV, in our population with mostly adequate vitamin B12 concentrations. These results support the role of folate in placental growth during early pregnancy.

Our results are largely in line with other studies reporting 1–12% differences in placental weight or growth measured after birth, in association with maternal folate concentrations or the timing of folic acid supplement use (Rolschau *et al.*, 1979; Timmermans *et al.*, 2009; Bergen *et al.*, 2012; Ren *et al.*, 2024). Especially considering the relative diminishing difference we observed over time, between 7 and 11 weeks of gestation, which could indicate an increase in the effect of other (maternal) factors on placental growth across gestation. In contrast, a systematic review reported no significant relationship between folic acid supplement use and placental weight; however, this might be due to the small sample size included in this systematic review and difficulties in measuring placental weight in an uniform manner (Fekete *et al.*, 2012). In mice, maternal dietary folate has been shown to increase placental weight (Zhang *et al.*, 2015; Zhou *et al.*, 2015). Another study found inhibited signaling of mTORC1 and mTORC2, stimulators of placental amino acid transport, in folate-deficient mice. This resulted in altered placental nutrient transport and lower fetal birth weight, but they reported no difference in placental weight (Rosario *et al.*, 2017).

The positive associations between maternal folate concentrations and early folic acid supplementation with first-trimester placental growth may be explained by the key role of folate in one-carbon metabolism. In one-carbon metabolism, folate provides methyl donors required for DNA synthesis and epigenetic processes including DNA and histone methylation (Friso *et al.*, 2017). For example, several studies have shown associations between measures of folate or folic acid and human placental DNA methylation of specific genes involved in growth and development (van Vliet *et al.*, 2024). Also, in *in vitro* studies, folic acid has been shown to affect DNA methylation, leading to an increased invasive potential of trophoblasts (Rahat *et al.*, 2022), and the apoptosis rate of human trophoblasts was higher when cultured without folate possibly due to impaired DNA synthesis and repair required for cell replication (Steegers-Theunissen *et al.*, 2000). Moreover, pregnancy (especially the first trimester) is characterized by epigenetic reprogramming and rapid cell replication and proliferation leading to an increased demand for methyl groups. Therefore, this period is considered a particularly vulnerable time span for epigenetic disruptions that can impair placental and fetal growth and development, and even health outcomes later in life (Steegers-Theunissen *et al.*, 2013).

Another potential underlying biological mechanism explaining the positive association between folate/folic acid and first-trimester placental growth could be the role of folate in the remethylation of homocysteine in the one-carbon metabolism (Froese *et al.*, 2019; Kaldygulova *et al.*, 2023). Higher plasma concentrations of maternal homocysteine have in turn been associated with smaller first-trimester placentas in our cohort (Hoek *et al.*, 2021) and with decreased placental weight postpartum by others (Bergen *et al.*, 2012). Moreover, hyperhomocysteinemia is associated with placental-mediated complications such as preeclampsia (Gaiday *et al.*, 2018). Elevated homocysteine concentrations are associated with oxidative stress, leading to impaired endothelial function which can negatively affect placentation and placental growth (Gaiday *et al.*, 2018; Yuan *et al.*, 2023). Folate may thus be protective against these processes by decreasing homocysteine concentrations and, as such, promote placental growth.

Serum folate concentrations are generally positively correlated with RBC folate concentrations (De Bruyn *et al.*, 2014) and are more often used in clinical settings, as they are easier to measure. However, RBC folate is a more reliable indicator of long-term folate status, while serum folate is more sensitive to recent folate intake (1–3 days), which likely has a smaller impact on overall placental growth. This may explain the less evident relationship found between serum folate and PV, with only significant larger PVs in women with Q3 as compared to Q1 serum folate concentrations.

We found no significant association between maternal serum vitamin B12 concentrations and placental growth. This is in line with another study in humans, which found no association between maternal vitamin B12 concentrations and postpartum placental weight (Bergen *et al.*, 2012). In contrast, smaller placentas have been found in vitamin B12 deficient mice (Sapehia *et al.*, 2023). However, vitamin B12 concentrations were adequate for the majority of the participants in our study population. Therefore, our results suggest that vitamin B12 concentrations do not affect first-trimester placental growth when vitamin B12 concentrations are sufficient. It would be of interest to investigate the relationship between vitamin B12 and placental growth in a population with a high prevalence of vitamin B12 deficiency. Notably, vitamin B12 deficiency could induce a functional folate deficiency, while folate concentrations could be normal or even high, which is referred to as the methyl-folate trap. This ‘trap’ is caused if the conversion from 5-methyl-tetrahydrofolate into tetrahydrofolate, in which vitamin B12 acts as cofactor, is hampered (Shane and Stokstad, 1985). In mice, oversupplementation with folic acid in vitamin B12 deficient mice, still resulted in smaller placental and fetal weights, which could be explained by a functional folate deficiency (Sapehia *et al.*, 2023). This should be taken into account when comparing our results to populations with high rates of vitamin B12 deficiency, and supports the need for further research on the associations between low vitamin B12 concentrations and placental development.

Importantly, excessive folic acid intake may also pose potential risks. Several studies have observed neurodevelopmental changes in the offspring of mice oversupplemented with folic acid. In humans, conflicting outcomes have been reported, although most studies have found positive associations with neurodevelopment in the offspring (reviewed in Xu *et al.*, 2024). In relation to placental weight, excessive supplementation of folic acid has been associated with a lower placental weight in rats (Shah *et al.*, 2017) and this trend was also observed in mice (Bahous *et al.*, 2017). Generally, the doses used in these studies largely exceeded the recommended daily intake of folate and were not representative of our study population. A previous study estimated that RBC folate concentrations above 1300 nmol/l had little additional benefit for the prevention of neural tube defects (Crider *et al.*, 2014). This supports the detrimental effects specifically of a folate deficiency, comparable to our results showing smaller PVs particularly in women with the lowest folate concentrations, while caution is required to prevent oversupplementation in humans.

Optimal first-trimester placental growth is not defined and a larger first-trimester placenta does not necessarily indicate a better functioning placenta. Yet, first-trimester placental growth is positively associated with embryonic and fetal growth (Reijnders *et al.*, 2021). Whether a larger placenta results in a larger embryo or whether their growths are independent of each other during the first trimester (largely before the feto-maternal circulation is established after unplugging of the spiral arteries), still remains to be elucidated. However, smaller first-trimester placental growth has been associated with several obstetric complications (Abdallah *et al.*, 2021; Park *et al.*, 2021), indicative of the positive effects of a larger first-trimester PV.

The strengths of our study include early measurements of PV using 3D ultrasounds enabling us to study the associations between first-trimester maternal folate and vitamin B12 concentrations directly with first-trimester growth, which has not been undertaken previously. We validated our results for RBC folate concentrations with serum folate concentrations, and in a larger cohort by investigating the association between the timing of folic acid supplement initiation and PV. These results demonstrate the robustness of our results. Limitations of our study are that, despite correcting for several confounders, potential residual confounding cannot be excluded because of the observational nature of this study. Also, the study was performed in a hospital-based setting, explaining the relatively high number of pregnancies conceived after IVF/ICSI and the high rates of preconceptional folic acid use. Moreover, vitamin B12 concentrations were adequate for the vast majority of our study population. Therefore, it would be of interest to investigate the studied associations in a population-based cohort as well as in populations with a high prevalence of vitamin B12 and/or folate deficiencies.

## Conclusions

We observed smaller first-trimester PVs particularly in women with the lowest RBC folate concentrations. As a validation, we show comparable results with smaller PVs in women with the lowest serum folate concentrations, and in women who initiated folic acid supplements following as compared to prior to conception. In our study population, with mostly adequate vitamin B12 serum concentrations, we found no associations between serum vitamin B12 concentrations and first-trimester placental growth. Our study emphasizes the importance of the preconceptional initiation of folic acid supplements to acquire adequate maternal folate concentrations, which can support placental growth during the first trimester.

## Supplementary Material

deaf095_Supplementary_Figure_S1

deaf095_Supplementary_Table_S1

## Data Availability

The data underlying this article will be shared on reasonable request to the corresponding author.
