# Supplementary material for: First-trimester maternal folate and vitamin B12 concentrations and their associations with first-trimester placental growth: the Rotterdam Periconception Cohort
Source: Hum Reprod. 2025 May 15;40(8):1485–94. doi: 10.1093/humrep/deaf095 (PMC12314151; doi:10.1093/humrep/deaf095)
Supplement: deaf095_Supplementary_Figure_S1 [file deaf095_supplementary_figure_s1.pdf]

OXFORD
human reproduction
Human Reproduction, 2025, 1–1
https://doi.org/10.1093/humrep/deaf095
Supplementary Data

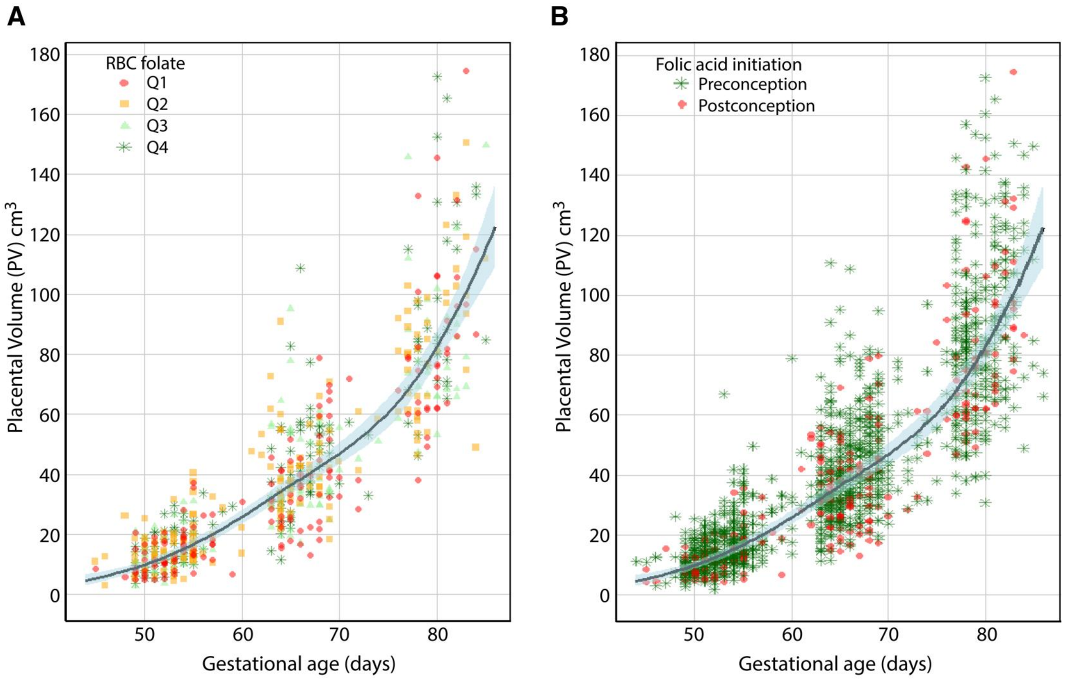

Supplemental Figure S1. Effect plots showing the placental volume (PV) trajectories during the first trimester of pregnancy for the total study population. (**A**) Individual data points based on red blood cell (RBC) folate Quartiles. (**B**) Individual data points based on the timing of folic acid supplement use initiation. Effect plots are based on model 2.
