# Supplementary material for: First-trimester maternal folate and vitamin B12 concentrations and their associations with first-trimester placental growth: the Rotterdam Periconception Cohort
Source: Hum Reprod. 2025 May 15;40(8):1485–94. doi: 10.1093/humrep/deaf095 (PMC12314151; doi:10.1093/humrep/deaf095)
Supplement: deaf095_Supplementary_Table_S1 [file deaf095_supplementary_table_s1.pdf]

OXFORD
human reproduction
Human Reproduction, 2025, 1–1
https://doi.org/10.1093/humrep/deaf095
Supplementary Data

Supplementary Table S1. Effect estimates for associations between first-trimester maternal serum folate concentrations and first-trimester placental volumes (PV $\sqrt[3]{cm^3}$).

	Serum folate			
	Model 1		Model 2	
	**Bèta (95% CI)**	**P-value**	**Bèta (95% CI)**	**P-value**
**Q1** (11.0–32.7 nmol/l) (n = 116)	Reference	Reference	Reference	Reference
**Q2** (32.8–39.2 nmol/l) (n = 117)	0.069 (−0.023 to 0.162)	0.142	0.071 (−0.028 to 0.170)	0.161
**Q3** (39.3–45.3 nmol/l) (n = 113)	0.104 (0.010–0.198)	0.030*	0.132 (0.027–0.237)	0.014*
**Q4** (45.4–141.6 nmol/l) (n = 114)	0.088 (−0.006 to 0.182)	0.066	0.082 (−0.023 to 0.187)	0.127

Quartile 1 is taken as reference. Model 1 is adjusted for gestational age at the ultrasound. Model 2 is additionally adjusted for fetal sex and maternal covariates age, geographic origin, education, periconceptional BMI, mode of conception, parity, and first-trimester vitamin B12 concentration. *Significance at $P \leq 0.05$.

© The Author(s) 2025. Published by Oxford University Press on behalf of European Society of Human Reproduction and Embryology.
This is an Open Access article distributed under the terms of the Creative Commons Attribution License (https://creativecommons.org/licenses/by/4.0/), which permits unrestricted reuse, distribution, and reproduction in any medium, provided the original work is properly cited.
Human Reproduction, 2025, 00, 1–12
https://doi.org/10.1093/humrep/deaf095
Original Article
